# The prevalence of MAFLD and its association with atrial fibrillation in a nationwide health check-up population in China

**DOI:** 10.3389/fendo.2022.1007171

**Published:** 2022-09-27

**Authors:** Fang Lei, Juan-Juan Qin, Xiaohui Song, Ye-Mao Liu, Ming-Ming Chen, Tao Sun, Xuewei Huang, Ke-Qiong Deng, Xiuran Zuo, Dongai Yao, Li-Juan Xu, Huiming Lu, Gang Wang, Feng Liu, Lidong Chen, Jie Luo, Jiahong Xia, Lin Wang, QiongYu Yang, Peng Zhang, Yan-Xiao Ji, Xiao-Jing Zhang, Zhi-Gang She, Qiang Zeng, Hongliang Li, Jingjing Cai

**Affiliations:** ^1^ Department of Cardiology, Renmin Hospital, School of Basic Medical Science, Wuhan University, Wuhan, China; ^2^ Institute of Model Animal, Wuhan University, Wuhan, China; ^3^ Department of Cardiology, Huanggang Central Hospital, Huanggang, China; ^4^ Huanggang Institute of Translational Medicine, Huanggang Central Hospital, Huanggang, China; ^5^ Department of Cardiology, Zhongnan Hospital of Wuhan University, Wuhan, China; ^6^ Department of Information, The Central Hospital of Wuhan, Wuhan, China; ^7^ Physical Examination Center, Zhongnan Hospital of Wuhan University, Wuhan, China; ^8^ Physical Examination Center, Renmin Hospital, Wuhan University, Wuhan, China; ^9^ General Medical Department, CR & WISCO General Hospital, Wuhan, China; ^10^ Basic Medical Laboratory, General Hospital of Central Theater Command, Wuhan, China; ^11^ Information Center, Hubei Provincial Hospital of Integrated Chinese and Western Medicine, Wuhan, China; ^12^ Department of Medical Examination Center, Taihe Hospital, Hubei University of Medicine, Shiyan, China; ^13^ Department of Neurosurgery, Taihe Hospital, Hubei University of Medicine, Shiyan, China; ^14^ Department of Cardiovascular Surgery, Union Hospital, Tongji Medical College, Huazhong University of Science and Technology, Wuhan, China; ^15^ Department of Hepatobiliary Surgery, Xi-Jing Hospital, Fourth Military Medical University, Xi’an, China; ^16^ Chinese Medicine Center, Shiyan Renmin Hospital, Shiyan, China; ^17^ Health Management Institute, The Second Medical Center & National Clinical Research Center for Geriatric Diseases, Chinese PLA General Hospital, Beijing, China; ^18^ Medical Science Research Center, Zhongnan Hospital of Wuhan University, Wuhan, China; ^19^ Department of Cardiology, The Third Xiangya Hospital, Central South University, Changsha, China

**Keywords:** mafld, insulin resistance, prevalence, atrial fibrillation, association

## Abstract

**Background and aims:**

The epidemiological characteristics of MAFLD and its relationship with atrial fibrillation (AF) are limited in China. Therefore, we explored the epidemiological characteristics of MAFLD from adults along with the association of MAFLD and 12-ECG diagnosed AF in a nationwide population from health check-up centers.

**Methods:**

This observational study used cross-sectional and longitudinal studies with 2,083,984 subjects from 2009 to 2017. Age-, sex-, and regional-standardized prevalence of MAFLD was estimated. Latent class analysis (LCA) was used to identify subclusters of MAFLD. Multivariable logistic regression and mixed-effects Cox regression models were used to analyze the relationship between MAFLD and AF.

**Results:**

The prevalence of MAFLD increased from 22.75% to 35.58% during the study period, with higher rates in males and populations with high BMI or resided in northern regions. The MAFLD population was clustered into three classes with different metabolic features by LCA. Notably, a high proportion of MAFLD patients in all clusters had overweight and prediabetes or diabetes. The MAFLD was significantly associated with a higher risk of AF in the cross-sectional study and in the longitudinal study. In addition, the coexistence of prediabetes or diabetes had the largest impact on subsequent AF.

**Conclusion:**

Our findings suggested a high prevalence of MAFLD and a high prevalence of other metabolic diseases in the MAFLD population, particularly overweight and glucose dysregulation. Moreover, MAFLD was associated with a significantly higher risk for existing and subsequent subclinical AF in the Chinese population.

## 1 Introduction

Nonalcoholic fatty liver disease (NAFLD) has become the most prevalent liver disease worldwide, with a whole range of changes in lifestyle, age structure, and social burdens (1, 2). Recent data have shown that many developing countries are bearing the fastest growth in the numbers of NAFLD cases. According to our recent meta-analysis, the estimated prevalence of NAFLD was 29.2% in China (3). The devastating complications of NAFLD are not confined to advanced liver disease but more commonly affect the cardiovascular system (4–8). Globally, 25-40% of NAFLD patients have cardiovascular diseases (CVDs), which is the leading cause of death in these individuals. Worryingly, CVD complications seem to be more prevalent in the Chinese population. Our previous meta-analysis demonstrated that approximately 55% of individuals with NAFLD have various forms of CVDs (3, 9).

Metabolic dysfunction-associated fatty liver disease (MAFLD) is an updated nomenclature for NAFLD that was developed in 2020 to overcome a diagnosis based on the exclusion of excessive alcohol use consumption and other forms of liver disease, which would accommodate the identification of a metabolically complicated fatty liver that is superimposed on chronic liver disease or alcoholism and would exclude a fatty liver that is unrelated to metabolic dysfunction (10). The shift in terminology is expected to positively impact diagnosis of the disease and sufficient intervention. Importantly, MAFLD criteria may be able to identify individuals at higher risk of CVD. Atrial fibrillation (AF) is the most common arrhythmia in clinical practice, which largely increased cardiovascular complication and mortality. Accumulating evidence showed that NAFLD is not only adversely correlated with atherosclerotic diseases, but also impacts cardiac electrical system (11). Numbers of studies have explored the correlation between NAFLD and the risk of both prevalent and incident AF, however, these have inconsistent results. A large prospective ongoing cohort based on the Netherland population showed that liver stiffness, and not fatty liver disease, was associated with higher prevalence of atrial fibrillation. It could be explained by AF-induced subclinical venous congestion drives this association (12). The evidence for these associations that is based on the new definition of MAFLD is still limited in the Chinese population. It has been reported that participants with MAFLD are more likely to have a higher risk of CVD than NAFLD participants in the non-Chinese population (13, 14). Therefore, we explored the association of MAFLD with AF in a large-scale population.

A routine physical examination can better detect chronic diseases, promote lifestyle changes, and control risk factors. Routine examinations have developed rapidly throughout the nation, and health examination institutions have spread from large to medium-sized cities. It has been reported that over 48 million (30.5%) individuals in the Chinese population had received health check-ups by 2019. Participation in the health examinations was voluntary. The statistics showed that approximately 63.3% of the participants were encouraged by their employers to undergo health examinations, which were offered free of charge (15). The data generated from health examinations provide a great opportunity to understand the epidemiological characteristics of numerous chronic diseases in the urban Chinese population.

Therefore, in this study, we first estimated the prevalence and trends of MAFLD in 2,083,984 individuals from nationwide health check-up centers. Latent class analysis was used to identify subclusters of individuals with different characteristics of MAFLD based on the six metabolic features. Then, we investigated the association between MAFLD and the prevalence and incidence of AF in the cross-sectional and the retrospective cohort datasets, respectively.

## 2 Materials and methods

### 2.1 Study design and participants

#### 2.1.1 Cross-sectional population

**Figure 1:**
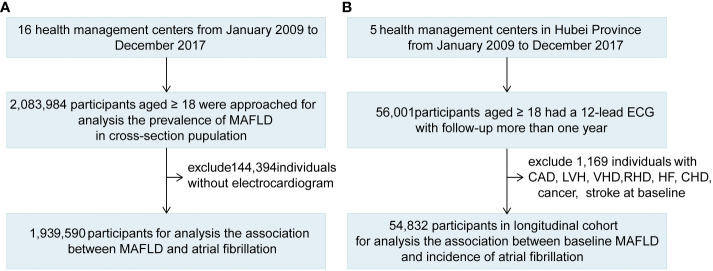
The Flowchart Showing the Strategy of Participant Enrollment. A schematic overview illustrating participants enrollment and the exclusion and inclusion criteria. **(A)** The flowchart of the cross-sectional study. **(B)** The flowchart of the cohort.

This study was designed as a multicenter, observational study comprising 2,083,984 adults from 16 health management centers from January 2009 to December 2017 (**Figure 1**; **Table S1**). The 16 health management centers from 10 provinces throughout the south and north (from 28.2 to 41.8°N latitude) in mainland China were included in our study, which covered three economic regions, including the eastern coastal zone, central zone, and western zone. Among these centers, 5 centers with 647,847 participants were located in northern China and 11 centers with 1436,137 participants were located in southern China. The participants who attended check-ups were a mixed population from nearby urban and rural areas. The population mainly consisted of adults with very diverse socioeconomic and occupational backgrounds, including public service employees, doctors, workers, farmers, and self-employed persons. Participation in the health examinations was on a voluntary basis, and some examinations were provided free of charge under the encouragement of the employer or the government. Among the cross-sectional population, 1,939,590 participants who had a 12-lead ECG were included to analyze the association between MAFLD and AF (**Figure 1**).

#### 2.1.2 Longitudinal cohort

We further explored the association between baseline MAFLD and the occurrence of AF in Hubei longitudinal cohorts. Individuals recruited in these cohorts had to meet two criteria (1): participants who underwent no less than two tests for 12-lead ECG between January 2009 and December 2017, with an interval of more than one year and (2) participants with a history of coronary heart disease (CAD), AF, left ventricle (LV) hypertrophy, valvular heart disease (VHD), rheumatic heart disease (RHD), heart failure (HF), congenital heart disease (CHD), cancer and stroke were excluded. The new-onset AF cohort with 54,832 participants was built to explore the association between baseline MAFLD and the incidence of AF during the follow-up (**Figure 1**).

The study protocol conforms to the ethical guidelines of the 1975 Declaration of Helsinki, was approved by the central ethics board of Renmin Hospital of Wuhan University and was accepted by the ethics center of each collaborating hospital. Patient informed consent was waived by the ethics committee from each hospital. Data on individual identification were removed, and only anonymous information remained.

### 2.2 Estimation of the prevalence of MAFLD

Age-, sex-, and regional-standardized prevalence was estimated in the overall population. It was estimated by using specific weights to represent the overall Chinese adult population aged 18 years or older for the subgroups from the sixth national census in 2010. The weights were calculated by the proportion of each age, sex, and region in the total population (16). In addition, the subgroup prevalence of sex, age, categories of BMI and location (northern/southern) were estimated.

### 2.3 Data collection

Each health screening center was equipped with professional and experienced medical professionals, and all participants underwent comprehensive anthropometric measurements and clinical examinations. Anthropometric measurements, including height, weight, waist circumference, hip circumference, systolic and diastolic blood pressures, and heart rate, were performed by well-trained physicians according to standard protocols. Body mass index (BMI) was calculated as weight divided by the square of height (kg/m^2^). After overnight fasting, the participants underwent routine blood tests, biochemical tests, and liver ultrasounds. Medical histories in the health examination records of each participant were collected. All imaging diagnoses were performed and evaluated by experienced imaging specialists at the medical health checkup center.

According to the BMI criteria for China, the participants were assigned to one of four groups: underweight (<18.5 kg/m^2^), normal (18.5-22.9 kg/m^2^), overweight (23.0-24.9 kg/m^2^), and obese (BMI ≥25.0 kg/m^2^) (17). T2DM was diagnosed according to personal history and the guidelines for the prevention and control of T2DM in China (2017) (18). Hypertension was based on personal history and the 2018 Chinese Guidelines for Prevention and Treatment of Hypertension (19). Metabolic syndrome (MetS) was defined based on the criteria from the CHPSNE study (20). Dyslipidemia was defined as self-reported dyslipidemia or the use of a lipid-lowering drug, according to the guidelines for the prevention and treatment of dyslipidemia in Chinese adults (21). Hyperuricemia was defined as the use of a uric acid-reducing drug or a serum uric acid level ≥420 mol/L in male and ≥360 mol/L in female (22). The triglyceride-glucose index (TyG index) is a marker of insulin resistance and was calculated using the formula TyG = Ln[FPG(mg/dL) × fasting triglyceride(mg/dL)/2] (23). The TyG threshold value for insulin resistance in Chinese adult individuals is 8.41 (24). The FIB-4 liver fibrosis score was calculated according to the existing equation incorporating age, AST level, ALT level, and platelets: (FIB-4 score = age [years] × AST [U/L])/([platelets (10^9^/L)] × (ALT [U/L])^1/2^)) (25). The estimated glomerular filtration rate (eGFR) was calculated according to the Modification of Diet in Renal Disease (MDRD) equations (26). CKD was defined as an eGFR < 60 ml/min per 1.73 m^2^ (CKD stage 3 or worse) (27). The primary outcome was AF diagnosed by 12-lead ECG (28).

### 2.4 Diagnosis of MAFLD

MAFLD was defined as the presence of both transabdominal ultrasound diagnosed FLD co-existing with any one of the following three conditions: overweight or obesity (defined as BMI ≥ 23 kg/m^2^); the presence of type 2 diabetes mellitus; lean or normal weight (BMI < 23 kg/m^2^) with the presence of metabolic dysregulation. Metabolic dysregulation was defined as the presence of at least two of the following metabolic risk abnormalities: 1) waist circumference ≥90 cm in male and ≥80 cm in female; 2) blood pressure ≥130/85 mmHg or the usage of specific drug treatment; 3) plasma triglycerides ≥1.70 mmol/L or the usage of specific drug treatment; 4) plasma high-density lipoprotein cholesterol ≥1.0 mmol/L in male and ≥1.3 mmol/L in female or the usage of specific drug treatment; and 5) prediabetes (fasting glucose level = 5.6-6.9 mmol/L or 2-hour postload glucose level = 7.8-11.0 mmol/L or HbA1c level = 5.7-6.4%) (29). The diagnosis of hepatic steatosis on ultrasound was based on the presence of hepatorenal echo contrast, liver parenchymal brightness, deep attenuation, and vascular blurring (30).

### 2.5 Latent class analysis

Latent class analysis (LCA) was used to identify subclusters of individuals with different characteristics of MAFLD based on the following metabolic features: 1) T2DM was diagnosed according to personal history and the guidelines for the prevention and control of T2DM in China; 2) prediabetes defined as insulin resistance (TyG >8.41 without T2DM) or prediabetes (fasting glucose level 5.6-6.9 mmol/L, or 2-hour postload glucose level = 7.8-11.0 mmol/L or HbA1c level = 5.7-6.4%); 3) overweight (BMI ≥23) or abdominal obesity (waist circumference ≥90 cm in male and ≥80 cm in female); 4) non-optimal blood pressure ≥130/85 mmHg or the usage of antihypertensive treatment was defined as high blood pressure; 5) dyslipidemia was defined as self-reported dyslipidemia, or the use of a lipid-lowering drug, or according to the guidelines for the prevention and treatment of dyslipidemia in Chinese adults (21); and 6) hyperuricemia (serum uric acid level ≥420 mol/L in male and ≥360 mol/L in female or using uric acid-reducing drugs). We explored several solutions, starting with two classes model and subsequently increased the number of classes up to a maximum of nine classes (**Table S2**, **Figure S1**). The best-fitting model was selected based on a combination of minimizing fit statistics and the interpretability of the emerging classes, specifically referring to the minimization of the Bayesian information criterion (BIC), the minimization of the Akaike information criterion (AIC), and the class solution where the log-likelihood plot started to level off (**Figures S1A-C**). Although the lowest AIC and BIC were achieved by the 9-class model (**Figures S1A, B**), when looking at the log-likelihood plot and the clinical interpretability of the emerging classes, the 3-class solution provided the optimal solution. Gains in model fit beyond the 3-class solution were minimal (**Figure S1C**) (31, 32).

### 2.6 Statistical analysis

R-3.6.3 (R Foundation for Statistical Computing, Vienna, Austria) was used to perform all statistical analyses. Comparisons between groups were performed with analysis of variance (ANOVA) for normally distributed variables, with the Mann–Whitney U test for nonparametric variables and with Fisher’s exact test or the chi-squared test for categorical variables.

Multivariable logistic regression models were used to analyze the relationship between MAFLD and AF. For variables that were partially missing, we used a mixed-type imputation method based on the R library MissForest to impute the missing data. A random forest model based on the rest of the variables in the dataset was constructed to predict the missing values with an estimation of the internally cross-validated errors (33, 34). After imputation, odds ratios (ORs) with 95% CIs were calculated accordingly. Model 1 was adjusted for age and sex, and Model 2 was additionally adjusted for self-reported smoking, self-reported drinking, red blood cell count, leukocyte count, hemoglobin count, platelet count, CAD, cancer, stroke, and CKD (stage ≥3).

Mixed-effects Cox regression models were used to calculate the hazard ratios (HRs) and 95% CIs for cohort. Model 1 was adjusted for age, sex, and medical center site as random effects. Model 2 was further adjusted for self-reported smoking, self-reported drinking, red blood cell count, leukocyte count, hemoglobin count, platelet count, and CKD (stage ≥3).

### 2.7 Sensitivity analyses

In the cohort study, we performed two sensitivity analyses to verify the robustness of the relationship of baseline MAFLD with the new-oneset AF. First, we estimated the relationship between baseline MAFLD and follow-up AF in the longitudinal cohort followed for more than 2 years. In the second sensitivity analysis, we further adjusted for FIB-4 scores in the mixed-effect Cox model to estimate the relationship between baseline MAFLD and follow-up AF.

## 3 Results

### 3.1 Baseline characteristics of the study population

The cross-sectional population comprised 2,083,984 Chinese adults. A total of 1,205,765 (57.86%) participants were male, and 878,219 (42.14%) were female (**Table 1**). The mean age was 44.96 (standard deviation [SD] 14.07) years. The baseline characteristics of the study participants are shown in **Table 1**. The mean BMI was 23.96 (SD 3.67) kg/cm^2^, the mean waist circumference was 82.92 (SD 10.61) cm, and the mean BP was 123 (SD 18)/77 (SD 12) mmHg. The data also revealed the average prevalence of other metabolic diseases at the national level in the study period, namely, T2DM, hypertension, MetS, dyslipidemia, and hyperuricemia. There were 130,361 (6.26%) individuals with T2DM, 510,953 (24.62%) with hypertension, 640,697 (32.66%) with MetS, 850,923 (43.01%) with dyslipidemia, and 244,479 (12.96%) with hyperuricemia (**Table 1**).

**Table 1:** Baseline Characteristics for the Cross-sectional Population.

	Total	Male	Female	*p value* ^†^	MAFLD	Non-MAFLD	*p value* ^†^
	N = 2083984	N = 1205765	N = 878219		N = 701718	N = 1382266	
Age (year, mean (SD))	44.96 (14.07)	44.99 (14.07)	44.91 (14.07)	<0.001	48.22 (12.60)	43.30 (14.48)	<0.001
Gender, Female, n (%)	878219 (42.14)	0 (0.00)	878219 (100.00)	<0.001	195880 (27.91)	682339 (49.36)	<0.001
BMI (kg/m^2^, mean (SD))	23.96 (3.67)	24.56 (3.52)	23.14 (3.72)	<0.001	26.82 (3.06)	22.51 (3.05)	<0.001
WC (cm, mean (SD))	82.92 (10.61)	87.31 (9.23)	76.38 (9.05)	<0.001	91.10 (8.15)	77.91 (8.64)	<0.001
SBP (mmHg, mean (SD))	123 (18)	126 (17)	120 (20)	<0.001	131 (18)	119 (17)	<0.001
DBP (mmHg, mean (SD))	77 (12)	79 (12)	74 (12)	<0.001	82 (12)	74 (11)	<0.001
Self-reported smoking, n (%)	123542 (5.93)	119389 (9.90)	4153 (0.47)	<0.001	60252 (8.59)	63290 (4.58)	<0.001
Self-reported drinking, n (%)	66905 (3.21)	60127 (4.99)	6778 (0.77)	<0.001	41620 (5.93)	25285 (1.83)	<0.001
FBG (mmol/L, mean (SD))	5.36 (1.26)	5.43 (1.35)	5.26 (1.13)	<0.001	5.79 (1.63)	5.14 (0.95)	<0.001
TC (mmol/L, mean (SD))	4.74 (1.03)	4.75 (1.10)	4.73 (0.94)	<0.001	4.99 (0.98)	4.61 (1.03)	<0.001
TG (mmol/L, mean (SD))	1.60 (1.42)	1.79 (1.59)	1.32 (1.08)	<0.001	2.29 (1.88)	1.22 (0.89)	<0.001
HDL-c (mmol/L, mean (SD))	1.35 (0.36)	1.27 (0.32)	1.47 (0.37)	<0.001	1.19 (0.29)	1.44 (0.36)	<0.001
LDL-c (mmol/L, mean (SD))	2.72 (0.81)	2.76 (0.79)	2.65 (0.82)	<0.001	2.92 (0.82)	2.61 (0.77)	<0.001
TBIL (μmol/L, mean (SD))	14.12 (6.08)	14.79 (6.33)	13.20 (5.58)	<0.001	13.95 (5.93)	14.21 (6.15)	<0.001
ALT (IU/L, mean (SD))	25.28 (22.79)	28.82 (25.24)	20.40 (17.80)	<0.001	34.06 (25.80)	20.79 (19.63)	<0.001
AST (IU/L, mean (SD))	23.35 (14.06)	24.34 (15.55)	22.04 (11.65)	<0.001	25.78 (14.18)	22.06 (13.82)	<0.001
BUN (mmol/L, mean (SD))	4.74 (1.34)	4.94 (1.36)	4.47 (1.26)	<0.001	4.94 (1.33)	4.64 (1.33)	<0.001
Creatinine (μmol/L, mean (SD))	72.29 (19.22)	79.37 (18.26)	62.56 (15.97)	<0.001	76.13 (17.85)	70.26 (19.60)	<0.001
Uric acid (μmol/L, mean (SD))	315.21 (90.45)	337.54 (91.61)	287.41 (80.78)	<0.001	361.21 (88.97)	294.24 (83.06)	<0.001
LEU (×10^9^/L, mean (SD))	6.30 (1.79)	6.47 (1.85)	6.06 (1.68)	<0.001	6.70 (1.70)	6.09 (1.80)	<0.001
RBC (×10^12^/L, mean (SD))	4.75 (0.56)	4.95 (0.56)	4.47 (0.42)	<0.001	4.92 (0.46)	4.66 (0.58)	<0.001
HGB (g/L, mean (SD))	144.12 (15.94)	151.42 (13.03)	134.01 (13.96)	<0.001	150.25 (14.09)	140.98 (15.92)	<0.001
PLT (×10^9^/L, mean (SD))	219.39 (55.23)	214.53 (53.50)	226.12 (56.86)	<0.001	222.23 (55.07)	217.93 (55.26)	<0.001
Type 2 diabetes, n (%)	130361 (6.26)	88216 (7.32)	42145 (4.80)	<0.001	89073 (12.69)	41288 (2.99)	<0.001
Hypertension, n (%)	510953 (24.62)	332548 (27.69)	178405 (20.41)	<0.001	281793 (40.31)	229160 (16.65)	<0.001
MetS, n (%)	640697 (32.66)	410492 (36.48)	230205 (27.51)	<0.001	428762 (65.82)	211935 (16.17)	<0.001
Dyslipidaemia, n (%)	850923 (43.01)	573330 (49.99)	277593 (33.38)	<0.001	450655 (65.55)	400268 (31.00)	<0.001
Hyperuricaemia, n (%)	244479 (12.96)	214232 (19.44)	30247 (3.86)	<0.001	153191 (23.32)	91288 (7.43)	<0.001
CAD, n (%)	29375 (1.41)	18582 (1.54)	10793 (1.23)	<0.001	13920 (1.98)	15455 (1.12)	<0.001
Cancer, n (%)	5978 (0.29)	2655 (0.22)	3323 (0.38)	<0.001	2243 (0.32)	3735 (0.27)	<0.001
Stroke, n (%)	6779 (0.33)	4029 (0.33)	2750 (0.31)	0.009	3033 (0.43)	3746 (0.27)	<0.001
FIB-4 (mean (SD))	1.11 (0.83)	1.11 (0.80)	1.12 (0.87)	0.006	1.10 (0.73)	1.12 (0.88)	<0.001
CKD, n (%)	27470 (1.43)	15354 (1.38)	12116 (1.49)	<0.001	11777 (1.77)	15693 (1.24)	<0.001

MAFLD, metabolic dysfunction-associated fatty liver disease; SD, standard deviation; BMI, body mass index; WC, waist circumference; SBP, systolic blood pressure; DBP, diastolic blood pressure; FBG, fasting blood glucose; TC, total cholesterol; TG, triglycerides; LDL-C, low-density lipoprotein cholesterol; HDL-C, high-density lipoprotein cholesterol; TBIL, total bilirubin; ALT, alanine aminotransferase; AST, aspartate transaminase; BUN, blood urea nitrogen; LEU, leukocyte count; RBC, red blood cell; HGB, haemoglobin; PLT, platelet count; MetS, metabolic syndrome; CAD, coronary heart disease; FIB-4, Fibrosis 4 Score; CKD, chronic kidney disease.

^†^P values were calculated by student’s t-test for normally distributed variables and the Wilcoxon rank-sum test for non-normal distributed variables, as well as the chi-square test or Fisher’s exact test for categorical variables.

The male and female participants were similar in age. The mean age was 44.99 (SD 14.07) years for males and 44.91 (SD 14.07) years for females. There were significant sex discrepancies in all biochemical indicators and medical history of diseases. Generally, the majority of metabolic indicators were significantly higher in males than in females, including BMI (24.56 kg/cm^2^ versus 23.14 kg/cm^2^), waist circumference (87.31 cm versus 76.38 cm), FBG (5.43 mmol/L versus 5.26 mmol/L), SBP (126 mmHg versus 120 mmHg), DBP (79 mmHg versus 74 mmHg), TGs (1.79 mmol/L versus 1.32 mmol/L), and LDL-c (2.76 mmol/L versus 2.65 mmol/L). In contrast, HDL-c level (1.27 mmol/L versus 1.47 mmol/L) was lower in males than in females (**Table 1**). The proportions of participants with metabolic diseases, including hypertension (27.69% versus 20.41%), MetS (36.48% versus 27.51%), dyslipidemia (49.99% versus 33.38%), and hyperuricemia (19.44% versus 3.86%), were significantly higher in male participants than in female participants (**Table 1**).

The participants with MAFLD were more likely to be male (72.09% versus 50.64%) (**Table 1**). The MAFLD group, in comparison with the non-MAFLD group, had a higher percentage of self-reported smokers (8.59% versus 4.58%) and had a higher BMI (26.82 kg/cm^2^ versus 22.51 kg/cm^2^), waist circumference (91.10 cm versus 77.91 cm), SBP (131 mmHg versus 119 mmHg), and DBP (82 mmHg versus 74 mmHg). Furthermore, FBG level (5.79 mmol/L versus 5.14 mmol/L), serum LDL-c level (2.92 mmol/L versus 2.61 mmol/L), and serum TG level (2.29 mmol/L versus 1.22 mmol/L) were significantly higher, and serum HDL-c level (1.19 mmol/L versus 1.44 mmol/L) was significantly lower in the MAFLD groups than in the non-MAFLD groups. The prevalence of complicating diseases was higher in participants with MAFLD, including hypertension, MetS, dyslipidemia, and hyperuricemia (**Table 1**). There were higher proportions of individuals with ECG-diagnosed AF (0.30% versus 0.25%) in the group of participants with MAFLD than in the group of those without MAFLD **(** **Table 2**).

### 3.2 The estimated prevalence of MAFLD and stratification by age, sex, and region

**Figure 2:**
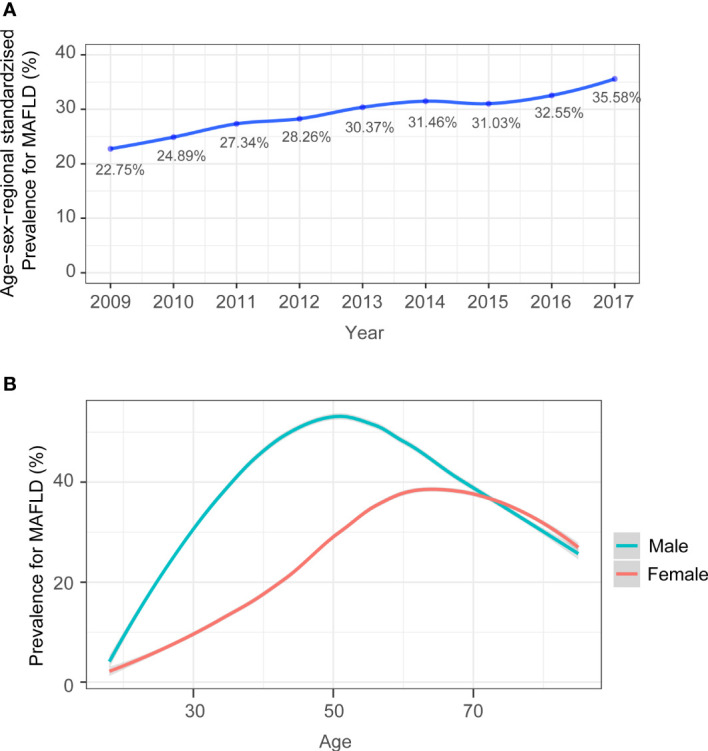
**(A)** Time trend in age, sex, and regional standardized prevalence of MAFLD. Age, sex, and regional standardized prevalence was estimated by using specific weights for subgroups from the sixth national census in 2010. **(B)** The age-specific prevalence (%) of MAFLD in males and females and in the total population were calculated, with 95% CIs represented by shaded regions.

The age, sex, and regional standardized prevalence of MAFLD increased from 22.75% in 2009 to 35.58% in 2017, with an average prevalence of 30.33% (95% CI, 30.27%-30.40%) (**Figure 2A**). The estimated prevalence of MAFLD was markedly higher in males (41.95%, 95% CI, 41.86%-42.04%) than in females (22.30%, 95% CI, 22.22%-22.39%). The prevalence of MAFLD was not linearly associated with age. The middle-age group (45 years ≤ age ≤ 64 years) had the highest prevalence of MAFLD (**Table 3**; **Figure 2B**). In males, the prevalence of MAFLD peaked at approximately 51 years of age and declined thereafter. A similar pattern was observed in females, with the prevalence peaking at approximately 66 years of age (**Figure 2B**). From 2009 to 2017, the prevalence of MAFLD increased in both sexes. However, the increase in MAFLD prevalence was significantly higher in males than in females. Moreover, for males, the most significant increase in prevalence occurred in the youngest age group, from 23.85% in 2009 to 47.75% in 2017. The largest increase for females was in the oldest age group (11.59% increase) (**Table 3**). Northern China also experienced a more rapid increase in MAFLD prevalence than southern China, with an increase by 21.76% versus 9.68%, respectively. The prevalence of MAFLD was increased in all BMI subgroups. From the underweight group (BMI<18.5) to the obese group (BMI ≥25), the prevalence increased from 0.25% (95% CI, 0.22-0.28) to 66.82% (95% CI, 66.71-66.92%) (**Table 3**).

**Table 3:** Prevalence of Metabolic Dysfunction-Associated Fatty Liver Disease among Overall Participants and Subgroups from 2009-2017.

MAFLD	2009	2010	2011	2012	2013	2014	2015	2016	2017		Overall
	n (%)	n (%)	n (%)	n (%)	n (%)	n (%)	n (%)	n (%)	n (%)		n (%)
	[95% CI]	[95% CI]	[95% CI]	[95% CI]	[95% CI]	[95% CI]	[95% CI]	[95% CI]	[95% CI]		[95% CI]
**Total **	20091 (22.75)	36972 (24.89)	48385 (27.34)	85893 (28.26)	111631 (30.37)	126489 (31.46)	129184 (31.03)	117449 (32.55)	25624 (35.58)		701718 (30.33)
	[22.45,23.05]	[24.66,25.13]	[27.13,27.56]	[28.09,28.43]	[30.21,30.53]	[31.31,31.61]	[30.88,31.18]	[32.39,32.71]	[35.19,35.96]		[30.27,30.40]
**MAFLD prevalence in sex subgroups**											
**Male**	12565 (29.24)	25382 (33.67)	34480 (35.69)	62388 (39.98)	80211 (42.93)	92845 (43.68)	93352 (43.74)	86360 (45.93)	18255 (53.81)	505838 (41.95)	
	[28.81,29.67]	[33.33,34.01]	[35.39,35.99]	[39.74,40.22]	[42.71,43.16]	[43.46,43.89]	[43.53,43.95]	[45.71,46.16]	[53.28,54.34]	[41.86,42.04]	
**Female**	7526 (23.26)	11590 (21.73)	13905 (21.46)	23505 (20.59)	31420 (22.67)	33644 (21.82)	35832 (22.62)	31089 (22.76)	7369 (28.56)	195880 (22.30)	
	[22.8,23.72]	[21.38,22.08]	[21.14,21.78]	[20.35,20.82]	[22.45,22.89]	[21.62,22.03]	[22.42,22.83]	[22.54,22.98]	[28.01,29.12]	[22.22,22.39]	
**MAFLD prevalence in age subgroups**											
**18≤Age ≤ 44**	7977 (20.67)	15004 (21.75)	19690 (22.56)	33035 (24.09)	44206 (26.63)	50366 (26.64)	50020 (26.81)	46135 (28.08)	8415 (32.75)	274848 (25.84)	
	[20.27,21.08]	[21.44,22.05]	[22.28,22.84]	[23.87,24.32]	[26.42,26.84]	[26.44,26.83]	[26.61,27.01]	[27.86,28.30]	[32.18,33.32]	[25.76,25.92]	
**45≤Age ≤ 64**	9331 (33.96)	18036 (37.84)	23611 (40.06)	43246 (40.89)	55959 (43.58)	64225 (44.27)	65417 (44.08)	60111 (45.42)	15167 (52.68)	355103 (43.15)	
	[33.40,34.52]	[37.41,38.28]	[39.67,40.46]	[40.59,41.18]	[43.31,43.85]	[44.01,44.52]	[43.82,44.33]	[45.15,45.69]	[52.1,53.26]	[43.05,43.26]	
**Age≥65**	2783 (30.00)	3932 (32.58)	5084 (33.49)	9612 (35.14)	11466 (36.96)	11898 (36.54)	13747 (37.35)	11203 (40.07)	2042 (38.98)	71767 (36.34)	
	[29.06,30.93]	[31.75,33.42]	[32.74,34.24]	[34.57,35.70]	[36.42,37.50]	[36.01,37.06]	[36.86,37.85]	[39.49,40.64]	[37.66,40.31]	[36.13,36.56]	
**MAFLD prevalence of age subgroups in male**											
**18≤Age ≤ 44**	5298 (23.85)	10974 (26.77)	14847 (28.20)	26216 (33.19)	34809 (36.81)	40993 (37.51)	40508 (38.02)	36991 (39.93)	6740 (47.75)		217376 (35.52)
	[23.29,24.41]	[26.34,27.20]	[27.82,28.59]	[32.86,33.51]	[36.50,37.11]	[37.22,37.79]	[37.73,38.31]	[39.62,40.25]	[46.93,48.58]		[35.40,35.64]
**45≤Age ≤ 64**	5684 (36.72)	12160 (43.73)	16685 (47.13)	30797 (49.90)	38797 (52.28)	45115 (53.13)	45111 (52.39)	42564 (54.12)	10456 (61.74)		247369 (51.40)
	[35.96,37.48]	[43.15,44.31]	[46.61,47.65]	[49.50,50.29]	[51.92,52.63]	[52.79,53.46]	[52.06,52.72]	[53.77,54.46]	[61.01,62.47]		[51.26,51.54]
**Age≥65**	1583 (29.97)	2248 (34.14)	2948 (34.44)	5375 (35.06)	6605 (36.62)	6737 (36.68)	7733 (37.26)	6805 (40.69)	1059 (36.82)		41093 (36.52)
	[28.73,31.21]	[33.00,35.29]	[33.43,35.45]	[34.31,35.82]	[35.92,37.32]	[35.98,37.38]	[36.60,37.92]	[39.95,41.44]	[35.06,38.58]		[36.24,36.81]
**MAFLD prevalence of age subgroups in female**											
**18≤Age ≤ 44**	2679 (16.37)	4030 (14.39)	4843 (13.98)	6819 (11.74)	9397 (13.15)	9373 (11.75)	9512 (11.89)	9144 (12.76)	1675 (14.46)		57472 (12.73)
	[15.80,16.93]	[13.98,14.80]	[13.62,14.35]	[11.47,12.00]	[12.91,13.40]	[11.52,11.97]	[11.66,12.11]	[12.52,13.00]	[13.82,15.11]		[12.63,12.82]
**45≤Age ≤ 64**	3647 (30.40)	5876 (29.59)	6926 (29.43)	12449 (28.26)	17162 (31.67)	19110 (31.76)	20306 (32.58)	17547 (32.68)	4711 (39.74)		107734 (31.53)
	[29.58,31.23]	[28.96,30.23]	[28.85,30.02]	[27.84,28.68]	[31.28,32.06]	[31.39,32.13]	[32.22,32.95]	[32.29,33.08]	[38.85,40.62]		[31.38,31.69]
**Age≥65**	1200 (30.03)	1684 (30.71)	2136 (32.26)	4237 (35.23)	4861 (37.43)	5161 (36.35)	6014 (37.48)	4398 (39.14)	983 (41.62)		30674 (36.10)
	[28.61,31.45]	[29.49,31.93]	[31.13,33.38]	[34.38,36.09]	[36.60,38.26]	[35.56,37.14]	[36.73,38.22]	[38.24,40.04]	[39.63,43.61]		[35.78,36.43]
**MAFLD prevalence in BMI subgroups**											
**BMI<18.5**	10 (0.29)	3 (0.05)	16 (0.19)	23 (0.18)	30 (0.21)	50 (0.30)	48 (0.31)	40 (0.30)	11 (0.49)		231 (0.25)
	[0.11,0.47]	[0.00,0.10]	[0.10,0.28]	[0.11,0.25]	[0.13,0.28]	[0.21,0.38]	[0.22,0.40]	[0.21,0.39]	[0.20,0.79]		[0.22,0.28]
**18.5≤BMI<23**	891 (3.13)	1215 (2.47)	1848 (3.02)	3460 (3.40)	5243 (4.48)	6260 (4.66)	6215 (4.72)	5437 (4.74)	1183 (6.18)		31752 (4.19)
	[2.93,3.33]	[2.34,2.61]	[2.88,3.15]	[3.29,3.51]	[4.36,4.59]	[4.55,4.78]	[4.60,4.83]	[4.62,4.86]	[5.84,6.52]		[4.15,4.24]
**23≤BMI<25**	4600 (26.35)	8049 (28.23)	10690 (30.44)	19539 (32.63)	25241 (34.59)	28999 (35.37)	28452 (33.92)	26872 (36.37)	5520 (41.06)		157962 (33.82)
	[25.70,27.00]	[27.71,28.75]	[29.96,30.92]	[32.26,33.01]	[34.25,34.94]	[35.05,35.70]	[33.60,34.24]	[36.02,36.71]	[40.22,41.89]		[33.68,33.95]
**BMI≥25**	14590 (56.25)	27705 (62.09)	35831 (63.39)	62871 (65.55)	81117 (67.07)	91180 (68.19)	94469 (67.08)	85100 (69.44)	18910 (75.94)		511773 (66.82)
	[55.65,56.86]	[61.64,62.54]	[62.99,63.78]	[65.25,65.85]	[66.80,67.33]	[67.94,68.44]	[66.83,67.32]	[69.18,69.70]	[75.41,76.47]		[66.71,66.92]
**MAFLD prevalence in region subgroups**											
**North**	5307 (24.11)	7481 (28.11)	14962 (32.75)	18028 (33.23)	41773 (36.54)	47359 (41.49)	49995 (38.47)	46229 (40.7)	17797 (45.87)		248931 (37.75)
	[23.54,24.67]	[27.57,28.65]	[32.32,33.18]	[32.83,33.63]	[36.26,36.82]	[41.21,41.78]	[38.21,38.74]	[40.41,40.99]	[45.37,46.36]		[37.64,37.87]
**South**	14784 (27.73)	29491 (28.88)	33423 (28.88)	67865 (31.42)	69858 (33.09)	79130 (31.32)	79189 (32.74)	71220 (33.75)	7827 (37.41)		452787 (31.78)
	[27.35,28.11]	[28.60,29.16]	[28.62,29.14]	[31.23,31.62]	[32.89,33.29]	[31.14,31.51]	[32.56,32.93]	[33.55,33.95]	[36.75,38.06]		[31.71,31.86]

CI, confidence interval; BMI, body mass index.

^†^Age-, sex-, regional-standardized prevalence was estimated by using specific weights for subgroups from the sixth national census in 2010.

### 3.3 The subclusters for MAFLD in Chinese patients

**Figure 3:**
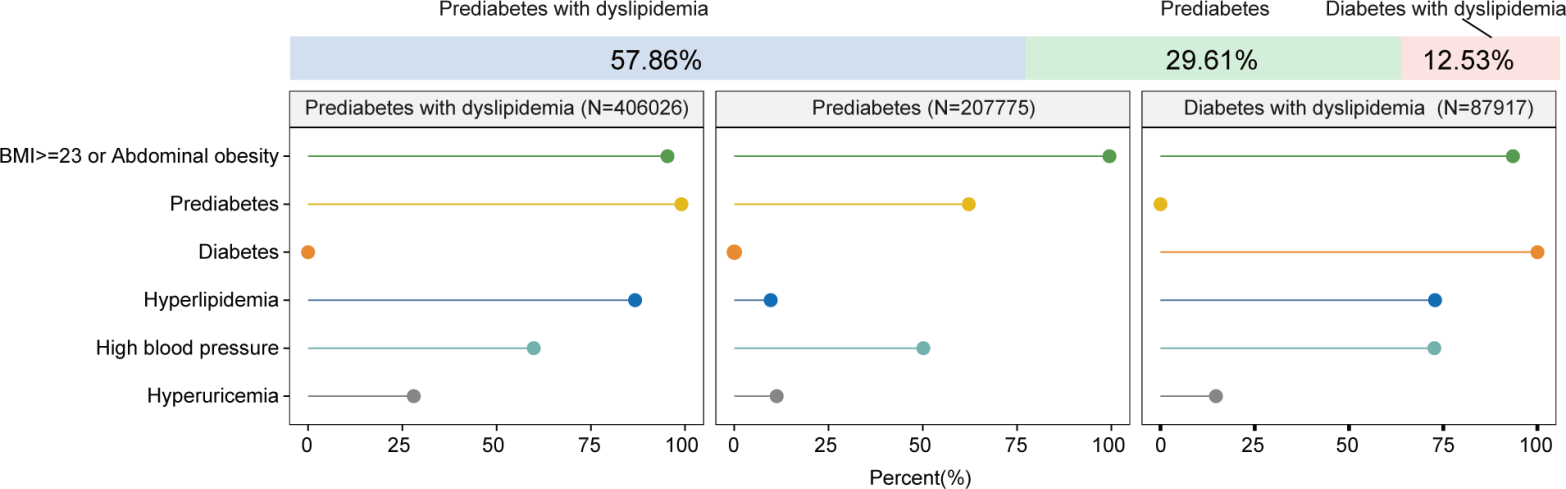
Probabilistic distribution of metabolic dysregulation in MAFLD subclusters.

LCA was performed to assimilate individuals with MAFLD into three clusters with characterized metabolic features. Notably, all clusters have overweight or abdominal obesity. Class 1 was named the prediabetes with dyslipidemia cluster, which accounts for 57.86% of the MAFLD patients. In addition to overweight or abdominal obesity, Class 1 was characterized by a high prevalence of prediabetes status, dyslipidemia and non-optimal blood pressure control or hypertension. Class 2 was defined the prediabetes cluster, which accounts for 29.61% of the MAFLD patients. This cluster has less metabolic comorbidities compared to the other two groups. Class 3 was termed the diabetes with dyslipidemia cluster, which was taken up to 12.53% of the MAFLD patients. This cluster was featured by the highest prevalence of metabolic comorbidities among three clusters, including diabetes, dyslipidemia and non-optimal blood pressure control or hypertension. The abovementioned clusters revealed that overweight and abdominal obesity may be the cornerstone for the development of MAFLD. In addition, prediabetes status was common in MAFLD population in China, which implies the necessity of screening prediabetes and insulin resistance before progressing to diabetic stage (**Figure 3**).

### 3.4 Association of MAFLD and AF in the cross-sectional population

In the cross-sectional dataset, the MAFLD group, showed a significantly higher prevalence of AF (0.30% versus 0.25%), in comparison with the non-MAFLD group (**Table 2**). In the logistic regression analysis, the ORs was 1.18 (95% CI 1.12-1.25, P < 0.001) for AF with MAFLD compared with those in the non-MAFLD group. After multivariable adjustment, the MAFLD group was associated with AF compared to the non-MAFLD group, with an OR of 1.12 (95% CI, 1.05-1.18; P < 0.001) (**Table 2**). Among MAFLD subclusters, the prevalence of AF in class 3, termed the diabetes with dyslipidemia cluster, was 0.55%, followed by 0.30% in class 2 and 0.24% in class 1 (**Table 2**). Class 3 had the highest correlations with the prevalence of AF compared with the non-MAFLD group or other MAFLD subgroups, including class 1 and class 2 (**Table 2**; **Table S3**).

**Table 2:** Association between MAFLD Group/Subclusters and Atrial Fibrillation in the Cross-sectional Analysis.

Groups	Prevalence of AF, n (%)	Odds ratio (95% confidence interval)			
		Model 1^†^	*p value* ^§^	Model 2^‡^	*p value* ^§^
**MAFLD versus non-MAFLD**					
Non-MAFLD	3245 (0.25)	Ref	–	Ref	–
MAFLD	1976 (0.30)	1.18 (1.12,1.25)	<0.001	1.12 (1.05,1.18)	<0.001
**MAFLD subgroups versus non-MAFLD**					
Non-MAFLD	3245 (0.25)	Ref	–	Ref	–
Class 1:Prediabetes with dyslipidemia	930 (0.24)	1.01 (0.94,1.09)	0.829	0.98 (0.90,1.05)	0.549
Class 2:Prediabetes	588 (0.30)	1.24 (1.13,1.35)	<0.001	1.25 (1.14,1.36)	<0.001
Class 3:Diabetes with dyslipidemia	458 (0.55)	1.69 (1.53,1.86)	<0.001	1.36 (1.23,1.51)	<0.001

**^†^** Model 1 the adjustment factors included age and sex.

**^‡^** Model 2 the adjustment factors included age, sex, self-reported smoking, self-reported drinking, red blood cell, leukocyte count, haemoglobin, platelet count, CAD, cancer, stroke and CKD.

**^§^** P values were calculated based on Logistic regression.

### 3.5 Association analysis of baseline MAFLD and the occurrence of AF in the longitudinal cohort

The baseline clinical characteristics for this longitudinal cohort from Hubei Province are shown in **Table S4**. During a median of 2.22 (IQR, 1.87-4.22) years of follow-up, there were 64(0.35%) participants developed AF in MAFLD population and 77 (0.21%) participants developed AF in non-MAFLD population (**Table 4**). Participants with MAFLD at baseline had a 1.84-fold increased risk of developing AF during the follow-up, and the HR was 1.84 (95% CI, 1.32-2.58, P < 0.001). After adjustment for potential confounders, the association still existed, with an HR of 1.99 (95% CI, 1.39-2.83, P < 0.001) (**Table 4**). In the longitudinal cohort, class 3 also had the highest incidence of AF (0.44%) (**Table 4**). Class1, Class 2 and 3 had increased risk of developing AF during the follow-up, with HRs 1.65 (95% CI, 1.04-2.60, P = 0.032), 2.49 (95% CI, 1.57-3.95, P < 0.001) and 2.09 (95% CI, 1.02-4.30, P = 0.045) (**Table 4**). However, due to the limited case of AF, the difference in AF incidences did not reach the significance threshold in subclasses (**Table S5**).

**Table 4:** The Association between Baseline MAFLD Group/Subclusters and the Incidence of Atrial Fibrillation in Longitudinal Cohort.

Groups	Incidence of AF,n (%)	Follow-up time (Year, median (IQR))	Hazards ratio (95% confidence interval)			
			Model 1^†^	*p value* ^§^	Model 2^‡^	*p value* ^§^
**MAFLD versus non-MAFLD**						
Non-MAFLD	77 (0.21)	2.22 (1.87,4.22)	Ref	–	Ref	–
MAFLD	64 (0.35)		1.84 (1.32,2.58)	<0.001	1.99 (1.39,2.83)	<0.001
**MAFLD subgroups versus non-MAFLD**						
Non-MAFLD	77 (0.21)	2.22 (1.87,4.22)	Ref	–	Ref	–
Class 1:Prediabetes with dyslipidemia	29 (0.29)		1.49 (0.96,2.30)	0.075	1.65 (1.04,2.60)	0.032
Class 2:Prediabetes	26 (0.44)		2.20 (1.40,3.44)	<0.001	2.49 (1.57,3.95)	<0.001
Class 3:Diabetes with dyslipidemia	9 (0.44)		2.17 (1.09,4.36)	0.029	2.09 (1.02,4.30)	0.045

**^†^** Model 1 the adjustment factors included age, sex and medical center as random effect.

**^‡^** Model 2 the adjustment factors included age, sex, self-reported smoking, self-reported drinking, red blood cell, leukocyte count, haemoglobin, platelet count, CKD and medical center as random effect.

**^§^** P values were calculated based on Mixed-effects Cox regression.

### 3.6 Sensitivity analysis

Sensitivity analyses were used to test the robustness of the results obtained from the longitudinal studies. First, we estimated the relationship between baseline MAFLD and the incidence of AF in a longitudinal cohort with more than 2 years of follow-up. MAFLD and MAFLD subclusters were repeatedly associated with the incidence of AF (**Table S6**).

Second, we further adjusted for FIB-4 scores in the mixed-effect Cox model to estimate the relationship between MAFLD and the incidence of AF in the longitudinal cohort. An positive association of MAFLD and MAFLD subclusters with the incidence AF exist (**Table S7**).

## 4 Discussion

In this nationwide study of 2,083,984 individuals from health check-up centers, we first estimated the epidemiological characteristics of MAFLD and explored the associated risk of MAFLD with the prevalence of AF in a cross-sectional population and the incidence of AF in retrospective cohorts. First, this study found that the standardized prevalence of MAFLD in urban Chinese adults was as high as 30.3%, and it increased significantly over the study period, from 22.75% in 2009 to 35.58% in 2017. Males and populations with an increased BMI and from northern regions of China had a markedly higher prevalence. Using LCA, the MAFLD population was clustered into three classes with different metabolic features. Notably, a high proportion of MAFLD patients with overweight and prediabetes or diabetes was seen in all clusters. Our data also showed that MAFLD was associated with 12-lead ECG diagnosed AF in the cross-sectional datasets and was associated with the incidence AF in the longitudinal cohort based on the individuals with routine health screening.

Our results revealed a heavy disease burden from MAFLD in the Chinese population with health screening. It rose rapidly by 12.83% during the study period, with a prevalence rate of 35.58% in 2017. The current prevalence of MAFLD approaches in many developed countries. For instance, the reported prevalence of MAFLD was 38.1% based on the 2017-2018 National Health and Nutrition Examination Survey (NHANES) database in the U.S (35). A total of 37.3% of Korean participants aged 40 to 64 years under routine health screening were diagnosed with MAFLD between 2009 and 2010 (14). Similar to other countries, the novel term “MAFLD” yields a higher prevalence rate when compared to the prevalence rate (29.2%) in a national meta-analysis in China (36). This novel term may explain the higher prevalence of MAFLD in individuals who have both metabolic disorders and excessive alcohol consumption or viral infections. Since accurate records for alcohol consumption have not been well documented in this large retrospective database, the concordance of the prevalence of MAFLD and NAFLD could not be analyzed.

According to our analysis, the prevalence of MAFLD increased significantly from 22.75% in 2009 to 35.58% in 2017. The growing national burden of MAFLD could be driven by economic developments and changes in lifestyle and nutritional patterns during the study period. In addition to the increased number of MAFLD patients in China, lifestyle transitions also lead to the increased prevalence of other metabolic diseases, e.g., dyslipidemia (43.1%), MetS (33.0%), hypertension (24.3%), hyperuricemia (13.0%) and self-reported diabetes (6.4%), which is in line with the rates from the most recent national studies (37–39).

Consistent with the trends in NAFLD reported in previous studies, our results confirm a higher prevalence of MAFLD in northern China than in southern China. In an effort to mitigate the risk and reduce the burden of disease in such a vast country, it is important to understand that the population risk varies geographically. First, geographical differences could be explained by temperature related different agricultural patterns (e.g., more maize, beans, and livestock in northern China) and associated socioeconomic structures and dietary patterns (northern China had lower vegetable intake, lower intake of seafood products and higher obesity and overweight rate), which lead to more general metabolic disturbances (40, 41). Next, compared with Han residents, NAFLD were more prevalent in the Hui, Uygur populations, Taiwan and the northwest region of mainland in China (9). The vast majority of these areas are in the north. At last, the GDP of southern China is significantly higher than that of northern China, and the GDP ranking was negatively correlated with the prevalence of NAFLD in China (3). Higher prevalence rates were reported among males than among females in China. In a randomized clinical trial suggests that combined hormone replacement therapy significantly decreased aminotransferase levels and presumed NAFLD compared in postmenopausal women with T2DM (42). This phenomenon might be related to the protective effect of estrogen on insulin resistance, lipid accumulation, hepatic VLDL secretion, lipotoxicity, and inhibition the activation of JNK and NF-кB in the progression of NAFLD (43–47). In addition, sex differences in obesity, other metabolic risk factors, and gut microbiome due to estrogen may further contribute to sex differences in NAFLD (48). Moreover, the peak MAFLD prevalence occurred earlier in males (approximately 51 years of age) than in females (approximately 66 years of age). This finding has also been reported (49–51). Potential reasons for the high prevalence in middle-aged males include sociocultural issues, life stress, and alcohol consumption in middle age. For females, an increased prevalence of FLD in elderly women may be associated with hormonal changes and higher susceptibility to metabolic risk factors after menopause (52, 53). Worryingly, this study revealed that the youngest age group (18 years ≤ age ≤ 44 years) had the fastest growth rate in the prevalence of MAFLD in males, while the oldest age group (aged ≥65 years) had the most rapid growth rate in the prevalence of MAFLD in females over time. This indicated that the young generation deserves special attention, as the accumulation of metabolic risk factors in early life would largely increase the risk of disease later in life. MAFLD has been considered a manifestation of multisystem metabolic dysfunction. Thus, we clustered MAFLD into different subclasses based on metabolic traits using LCA. Of note, overweight and prediabetes or diabetes were the main characteristic of all MAFLD clusters.

AF is the most common form of cardiac arrhythmia with severe cardiac consequences. Although the majority of cross-sectional studies revealed that NAFLD is associated with a markedly higher prevalence of AF, the evidence from longitudinal studies is more controversial (54–61). A longitudinal study with the Framingham Heart Study Offspring and Third Generation Cohort participants showed that liver fat by computed tomography scan was not significantly associated with an increased prevalence or incidence of AF over 12 years of follow-up (61). A large prospective ongoing cohort within the Rotterdam Study showed fatty liver disease was not associated with prevalent or incident atrial fibrillation; while liver stiffness was significantly associated with AF, especially among those without steatosis (12). We observed an association between MAFLD and AF in the cross-sectional study. Meantime, baseline MAFLD was also associated with the incidence of AF during follow-up. Although there is still some controversy about the relationship between MAFLD and AF, three meta-analyses suggested that NAFLD was associated with an increased risk of AF (11, 62, 63). Our finding is consistent with the results from Targher G’s meta-analysis that NAFLD with T2DM had the highest risk of developing AF in NAFLD (11). This was probably because T2DM patients had a greater propensity for ectopic and visceral fat deposition and a higher level of pro-inflammatory, profibrogenic, and vasoactive mediators, which facilitated the development of AF (64, 65). The discrepancies in the studies reflect the heterogeneous patients composing a broad spectrum of disease severity and potential complications. The discrepancies in the studies reflect the heterogeneous patients composing differences in genetic background, disease severity, potential complications and measurement tools for assessment of liver fat contents. We noticed that the studies based on the Western population, for example, the Framingham Heart Study population and the Rotterdam Study population, showed a weak association between liver fat and AF (12, 61). However, the studies from the Asian population showed positive associations between liver fat and AF (56, 57). Thus, we postulated genetic predisposition of AF might exist in a population with fatty liver disease in the Asian population. Further, we included the MAFLD population instead of NAFLD in this study. The difference in metabolic characteristics and disease stages of the population may bring such discrepancies (56, 66). Last, different methodologies were applied for the measurement of liver disease, which could be an important factor attributed to the differences in the results. Patients should be staged and stratified more precisely based on their genetic background, imaging or histological characteristics and comorbidities in further studies. In addition, to obtain precise and solid evidence regarding the relationship between MAFLD and AF or CVD complications, large prospective longitudinal studies of MAFLD need to be designed. Meantime, a longitudinal study should be carried out for an extended period of time to observe sufficient CVD events associated with AF.

The limitations of the present study merit attention. First, the study populations were based on national health examinations and were not based on random sampling, and the study data may underrepresent the rural populations of China. Second, the survey on alcohol consumption was not thoroughly conducted in a large population. Third, due to limitations in the methodology (ultrasound) for MAFLD examination that was applied in routine health check-ups, individuals could not be stratified by the severity of liver injury. Fourth, the number of missing values and imputations may also lead to inevitable bias in the results. Fifth, a causal relationship between MAFLD and cardiac arrhythmia could not be derived owing to the retrospective study design. Sixth, the limited number of participants in the MAFLD subclasses and the short follow-up period may result in an insignificant correlation between MAFLD and the incidence of AF and its associated cardiovascular outcomes.

## 5 Conclusion

This study adds knowledge of the epidemiological features of MAFLD based on health check-ups in China using a cross-sectional study with a total of 2,083,984 individuals. MAFLD patients were clustered into three subgroups with different metabolic features, with a high proportion of MAFLD patients developing overweight and prediabetes or diabetes in all clusters. Furthermore, this study revealed that MAFLD is associated with a significantly higher risk for the prevalence of AF in cross-sectional populations and the incidence of AF in longitudinal cohorts based on this real-world data.

## Data availability statement

The original contributions presented in the study are included in the article/Supplementary Material. Further inquiries can be directed to the corresponding authors.

## Ethics statement

The studies involving human participants were reviewed and approved by Ethics board of Renmin Hospital of Wuhan University. Written informed consent for participation was not required for this study in accordance with the national legislation and the institutional requirements.

## Author contributions

FL, J-JQ, XS and Y-ML designed the study, collected and analyzed data and drafted the manuscript. M-MC, TS, XH and K-QD performed the statistical analysis and interpreted data. XZ, DY, L-JX, HL, GW, FLi, LC, JL, JX, LW and QY assisted in data collection. PZ, Y-XJ, X-JZ and Z-GS performed critical revision of the manuscript for important intellectual content. QZ, JC and HLL conceived and supervised the study, critical revision of the manuscript for important intellectual content, and supervised the study. All authors contributed to the article and approved the submitted version.

## Funding

This work was supported by the National Science Foundation of China [81870171, 82170595, 82170436, 82000299, 82000386], the National Key R&D Program of China [2020YFC2004702], Henan Charity Federation Hepatobiliary Fund [GDXZ2021008], Medical flight plan of Wuhan University [TFJH2018006], and Excellent Doctoral Program of Zhongnan Hospital of Wuhan University [ZNYB2019001].

## Acknowledgments

We thank the Supercomputing Center of Wuhan University for the numerical calculations in this paper.

## Conflict of interest

The authors declare that the research was conducted in the absence of any commercial or financial relationships that could be construed as a potential conflict of interest.

## Publisher’s note

All claims expressed in this article are solely those of the authors and do not necessarily represent those of their affiliated organizations, or those of the publisher, the editors and the reviewers. Any product that may be evaluated in this article, or claim that may be made by its manufacturer, is not guaranteed or endorsed by the publisher.

## Abbreviations

MAFLD, metabolic (dysfunction)-associated fatty liver disease; MetS, Metabolism syndrome; BMI, Body mass index; NAFLD, Nonalcoholic fatty liver disease; T2DM, Type 2 diabetes; FBG, Fasting blood glucose; BP, Blood pressure; SBP, Systolic blood pressure; DBP, Diastolic blood pressure; TC, Total cholesterol; TG, Triglycerides; HDL-c, High-density lipoprotein cholesterol; LDL-c, Low-density lipoprotein cholesterol; TBIL, Total bilirubin; DBIL, Direct Bilirubin; ALT, Alanine; aminotransferase; AST, Aspartate transaminase; AF, Atrial fibrillation; BUN, Blood urea nitrogen; WBC, White blood cell; RBC, Red blood cell; HGB, Haemoglobin; PLT, Platelet count; FIB-4, Fibrosis 4 Score; CKD, chronic kidney disease; SD, Standard deviation; LAC, Latent class analysis; ORs, Odd ratios; HR, hazard ratios.
