# Supplementary material for: The prevalence of MAFLD and its association with atrial fibrillation in a nationwide health check-up population in China
Source: Front Endocrinol (Lausanne). 2022 Sep 27;13:1007171. doi: 10.3389/fendo.2022.1007171 (PMC9551383; doi:10.3389/fendo.2022.1007171)
Supplement: Supplementary file 1 [file DataSheet_1.docx]

**Supplemental information:** **The Prevalence of MAFLD and its Association with Atrial Fibrillation in a Nationwide Health Check-Up Population in China**

Fang Lei^*^, Juan-Juan Qin^*^, Xiaohui Song^*^, Ye-Mao Liu^*^, Ming-Ming Chen, Tao Sun, Xuewei Huang, Ke-Qiong Deng, Xiuran Zuo, Yao Dongai, Li-Juan Xu, Huiming Lu, Gang Wang, Feng Liu, Lidong Chen, Jie Luo, Jiahong Xia, Lin Wang, QiongYu Yang, Peng Zhang, Yan‑Xiao Ji, Xiao-Jing Zhang, Zhi-Gang She, Qiang Zeng^†^, Hongliang Li^†^, Jingjing Cai^†^

**Supplemental Table list:**

**Table S1. Beginning and Ending Dates for Data Collection in 16 Health Management Centers**

**Table S2. Fit statistics for latent class models by the number of classes**

**Table S3. The Association between MAFLD Subclusters and and Atrial Fibrillation in the Cross-sectional Analysis**

**Table S4. Baseline Characteristics of the Atrial Fibrillation Cohort**

**Table S5. The Association between Baseline MAFLD Subclusters and the Incidence of Atrial Fibrillation in Longitudinal Cohort**

**Table S6. Sensitivity Test I for the Incidence of Atrial Fibrillation in Baseline MAFLD Group/Subclusters versus Baseline Non-MAFLD Group during follow-up time above 2 years**

**Table S7. Sensitivity Test II for the Incidence of Atrial Fibrillation in Baseline MAFLD Group/Subclusters versus Baseline Non-MAFLD Group after Further adjusting FIB-4**

**Figure S1. Scree-like plots of fit statistics (Bayesian Information Criteria (BIC), Akaike Information Criteria (AIC), the log-likelihood (LLIK)) for latent class models by the number of classes**

**Table S1. Beginning and Ending Dates for Data Collection in 16 Health Management Centers**

| **Center** | **Region** | **Beginning date** | **Ending date** |
| --- | --- | --- | --- |
| C01 | North | 2009.01.01 | 2017.12.31 |
| C02 | North | 2011.01.01 | 2017.12.31 |
| C03 | North | 2014.01.01 | 2017.12.31 |
| C04 | North | 2013.01.01 | 2017.12.31 |
| C05 | North | 2011.01.01 | 2017.12.31 |
| C06 | South | 2009.01.01 | 2017.12.31 |
| C07 | South | 2009.01.01 | 2016.12.31 |
| C08 | South | 2012.01.01 | 2015.12.31 |
| C09 | South | 2010.01.01 | 2016.12.31 |
| C10 | South | 2014.01.01 | 2016.12.31 |
| C11 | South | 2009.01.01 | 2016.12.31 |
| C12 | South | 2009.01.01 | 2016.12.31 |
| C13 | South | 2010.01.01 | 2016.12.31 |
| C14 | South | 2012.01.01 | 2017.12.31 |
| C15 | South | 2009.01.01 | 2017.12.31 |
| C16 | South | 2012.01.01 | 2016.12.31 |

**Table S2. Fit statistics for latent class models by the number of classes**

| **Class** | **BIC** | **AIC** | **Log Likelihood** |
| --- | --- | --- | --- |
| 2 | 3777080 | 3776931 | -1888453 |
| 3 | 3687851 | 3687622 | -1843791 |
| 4 | 3685639 | 3685329 | -1842638 |
| 5 | 3683428 | 3683038 | -1841485 |
| 6 | 3683021 | 3682551 | -1841235 |
| 7 | 3682909 | 3682359 | -1841131 |
| 8 | 3682972 | 3682341 | -1841116 |
| 9 | 3681413 | 3680702 | -1840289 |

**Abbreviations:** BIC, bayesian information criterion; AIC, Akaike information criterion.

**Table S3. The Association between MAFLD Subclusters and and Atrial Fibrillation in the Cross-sectional Analysis**

| **Groups** | **Odds ratio**  **(95% confidence interval)^†^** | ***p value*^‡^** |
| --- | --- | --- |
| **Class 1 versus Class 2** | | |
| Class 1:Prediabetes with dyslipidemia | Ref | - |
| Class 2:Prediabetes | 1.23(1.10,1.36) | <0.001 |
| **Class 2 versus Class 3** | | |
| Class 2:Prediabetes | Ref | - |
| Class 3:Diabetes with dyslipidemia | 1.18(1.03,1.34) | 0.014 |
| **Class 1 versus Class 3** | | |
| Class 1:Prediabetes with dyslipidemia | Ref | - |
| Class 3:Diabetes with dyslipidemia | 1.55(1.38,1.74) | <0.001 |

**^†^** The adjustment factors included age, sex, self-reported smoking, self-reported drinking, red blood cell, leukocyte count, haemoglobin, platelet count, CAD, cancer, stroke and CKD.

**^‡^** P values were calculated based on Logistic regression.

**Table S4. Baseline Characteristics of the Atrial Fibrillation Cohort**

|  | **Total** | **MAFLD** | **Non-MAFLD** | ***p value*^†^** |
| --- | --- | --- | --- | --- |
|  | **N=54,832** | **N=18,135** | **N=36,697** |  |
| Age (year, mean (SD)) | 46.44(13.57) | 49.06(12.28) | 45.14(13.98) | <0.001 |
| Gender, Female, n(%) | 20169(36.78) | 4205(23.19) | 15964(43.50) | <0.001 |
| BMI (kg/m^2^, mean (SD)) | 23.66(3.26) | 26.42(2.66) | 22.30(2.59) | <0.001 |
| WC (cm, mean (SD)) | 80.99(9.94) | 88.38(7.93) | 76.98(8.52) | <0.001 |
| SBP (mmHg, mean (SD)) | 120(17) | 127(17) | 116(17) | <0.001 |
| DBP (mmHg, mean (SD)) | 76(11) | 81(11) | 73(10) | <0.001 |
| self-reported smoking, n(%) | 9066(16.53) | 3896(21.48) | 5170(14.09) | <0.001 |
| self-reported drinking, n(%) | 2472(4.51) | 1231(6.79) | 1241(3.38) | <0.001 |
| FBG (mmol/L, mean (SD)) | 5.38(1.20) | 5.76(1.58) | 5.19(0.90) | <0.001 |
| TC (mmol/L, mean (SD)) | 4.69(0.92) | 4.97(0.95) | 4.55(0.87) | <0.001 |
| TG (mmol/L, mean (SD)) | 1.50(1.35) | 2.22(1.80) | 1.15(0.86) | <0.001 |
| HDL-c (mmol/L, mean (SD)) | 1.29(0.31) | 1.16(0.25) | 1.36(0.32) | <0.001 |
| LDL-c (mmol/L, mean (SD)) | 2.75(0.77) | 2.95(0.81) | 2.64(0.74) | <0.001 |
| TBIL (μmol/L, mean (SD)) | 13.67(5.99) | 13.82(5.95) | 13.60(6.01) | 0.001 |
| ALT (IU/L, mean (SD)) | 25.23(21.82) | 34.01(24.24) | 20.87(19.07) | <0.001 |
| AST (IU/L, mean (SD)) | 24.34(15.95) | 27.19(13.39) | 23.07(16.81) | <0.001 |
| BUN (mmol/L, mean (SD)) | 4.99(1.30) | 5.12(1.24) | 4.93(1.32) | <0.001 |
| Creatinine (μmol/L, mean (SD)) | 70.88(17.51) | 73.59(15.83) | 69.54(18.14) | <0.001 |
| Uric acid (μmol/L, mean (SD)) | 312.78(86.51) | 351.88(87.39) | 292.62(78.80) | <0.001 |
| LEU (×10^9^/L, mean (SD)) | 6.22(1.57) | 6.72(1.59) | 5.98(1.51) | <0.001 |
| RBC (×10^12^/L, mean (SD)) | 4.69(0.50) | 4.84(0.46) | 4.61(0.50) | <0.001 |
| HGB (g/L, mean (SD)) | 144.20(15.14) | 149.71(13.27) | 141.47(15.26) | <0.001 |
| PLT (×10^9^/L, mean (SD)) | 215.14(53.55) | 219.47(53.47) | 212.99(53.46) | <0.001 |
| Type 2 diabetes, n(%) | 3128(5.70) | 2054(11.33) | 1074(2.93) | <0.001 |
| Hypertension, n(%) | 13279(24.35) | 7219(40.04) | 6060(16.60) | <0.001 |
| MetS, n(%) | 13605(28.30) | 9387(62.18) | 4218(12.79) | <0.001 |
| Dyslipidaemia, n(%) | 25395(47.03) | 12832(71.33) | 12563(34.88) | <0.001 |
| Hyperuricaemia, n(%) | 6502(12.19) | 3947(22.33) | 2555(7.17) | <0.001 |
| FIB-4 ( mean (SD)) | 1.22(0.77) | 1.18(0.71) | 1.24(0.79) | <0.001 |
| CKD, n(%) | 529(1.01) | 193(1.10) | 336(0.96) | 0.115 |

Abbreviations: MAFLD, metabolic dysfunction-associated fatty liver disease; SD, standard deviation; BMI, body mass index; WC, waist circumference; SBP, systolic blood pressure; DBP, diastolic blood pressure; FBG, fasting blood glucose; TC, total cholesterol; TG, triglycerides; LDL-C, low-density lipoprotein cholesterol; HDL-C, high-density lipoprotein cholesterol; TBIL, total bilirubin; ALT, alanine aminotransferase; AST, aspartate transaminase; BUN, blood urea nitrogen; LEU, leukocyte count; RBC, red blood cell; HGB, haemoglobin; PLT, platelet count; MetS, metabolic syndrome; FIB-4, Fibrosis 4 Score; CKD, chronic kidney disease.

**^†^** P values were calculated by student’s t-test for normally distributed variables and the Wilcoxon rank-sum test for non-normal distributed variables, as well as the chi-square test or Fisher’s exact test for categorical variables

**Table S5. The Association between Baseline MAFLD Subclusters and the Incidence of Atrial Fibrillation in Longitudinal Cohort**

| **Groups** | **Hazards ratio**  **(95% confidence interval)^†^** | ***p value*^‡^** |
| --- | --- | --- |
| **Class 1 versus Class 2** | | |
| Class 1:Prediabetes with dyslipidemia | Ref | - |
| Class 2:Prediabetes | 1.50(0.87,2.57) | 0.144 |
| **Class 2 versus Class 3** | | |
| Class 2:Prediabetes | Ref | - |
| Class 3:Diabetes with dyslipidemia | 0.78(0.35,1.74) | 0.544 |
| **Class 1 versus Class 3** | | |
| Class 1:Prediabetes with dyslipidemia | Ref | - |
| Class 3:Diabetes with dyslipidemia | 1.27(0.59,2.75) | 0.537 |

**^†^** The adjustment factors included age, sex, self-reported smoking, self-reported drinking, red blood cell, leukocyte count, haemoglobin, platelet count, CKD and medical center as random effect.

**^‡^** P values were calculated based on Mixed-effects Cox regression.

**Table S6. Sensitivity Test I for the Incidence of Atrial Fibrillation in Baseline MAFLD Group/Subclusters versus Baseline Non-MAFLD Group during follow-up time above 2 years**

| **Groups** | **Incidence of AF,**  **n(%)** | **Follow-up time**  **(Year, median (IQR))** | **Hazard ratio**  **(95% confidence interval)^†^** | ***p value*^‡^** |
| --- | --- | --- | --- | --- |
| **MAFLD versus Non-MAFLD** | | | | |
| Non-MAFLD | 46(0.19) | 3.69(2.19,5.10) | Ref | - |
| MAFLD | 43(0.34) |  | 2.29(1.47,3.57) | <0.001 |
| **MAFLD subgroups versus Non-MAFLD** | | | | |
| Non-MAFLD | 46(0.19) | 3.69(2.19,5.10) | Ref | - |
| Class 1:Prediabetes with dyslipidemia | 19(0.27) |  | 1.89(1.07,3.34) | 0.029 |
| Class 2:Prediabetes | 18(0.44) |  | 2.73(1.55,4.81) | <0.001 |
| Class 3:Diabetes with dyslipidemia | 6(0.41) |  | 2.53(1.04,6.15) | 0.041 |

**^†^** The adjustment factors included age, sex, self-reported smoking, self-reported drinking, red blood cell, leukocyte count, haemoglobin, platelet count, CKD and medical center as random effect.

**^‡^** P values were calculated based on Mixed-effects Cox regression.

**Table S7. Sensitivity Test II for the Incidence of Atrial Fibrillation in Baseline MAFLD Group/Subclusters versus Baseline Non-MAFLD Group after Further adjusting FIB-4**

| **Groups** | **Hazard ratio**  **(95% confidence interval)^†^** | ***p value*^‡^** |
| --- | --- | --- |
| **MAFLD versus Non-MAFLD** | | |
| Non-MAFLD | Ref | - |
| MAFLD | 2.02(1.42,2.88) | <0.001 |
| **MAFLD subgroups versus Non-MAFLD** | | |
| Non-MAFLD | Ref | - |
| Class 1:Prediabetes with dyslipidemia | 1.65(1.04,2.60) | 0.032 |
| Class 2:Prediabetes | 2.52(1.59,4.00) | <0.001 |
| Class 3:Diabetes with dyslipidemia | 2.22(1.08,4.56) | 0.031 |

**^†^** The adjustment factors included age, sex, self-reported smoking, self-reported drinking, red blood cell, leukocyte count, haemoglobin, platelet count, CKD, FIB-4 and medical center as random effect.

**^‡^** P values were calculated based on Mixed-effects Cox regression.


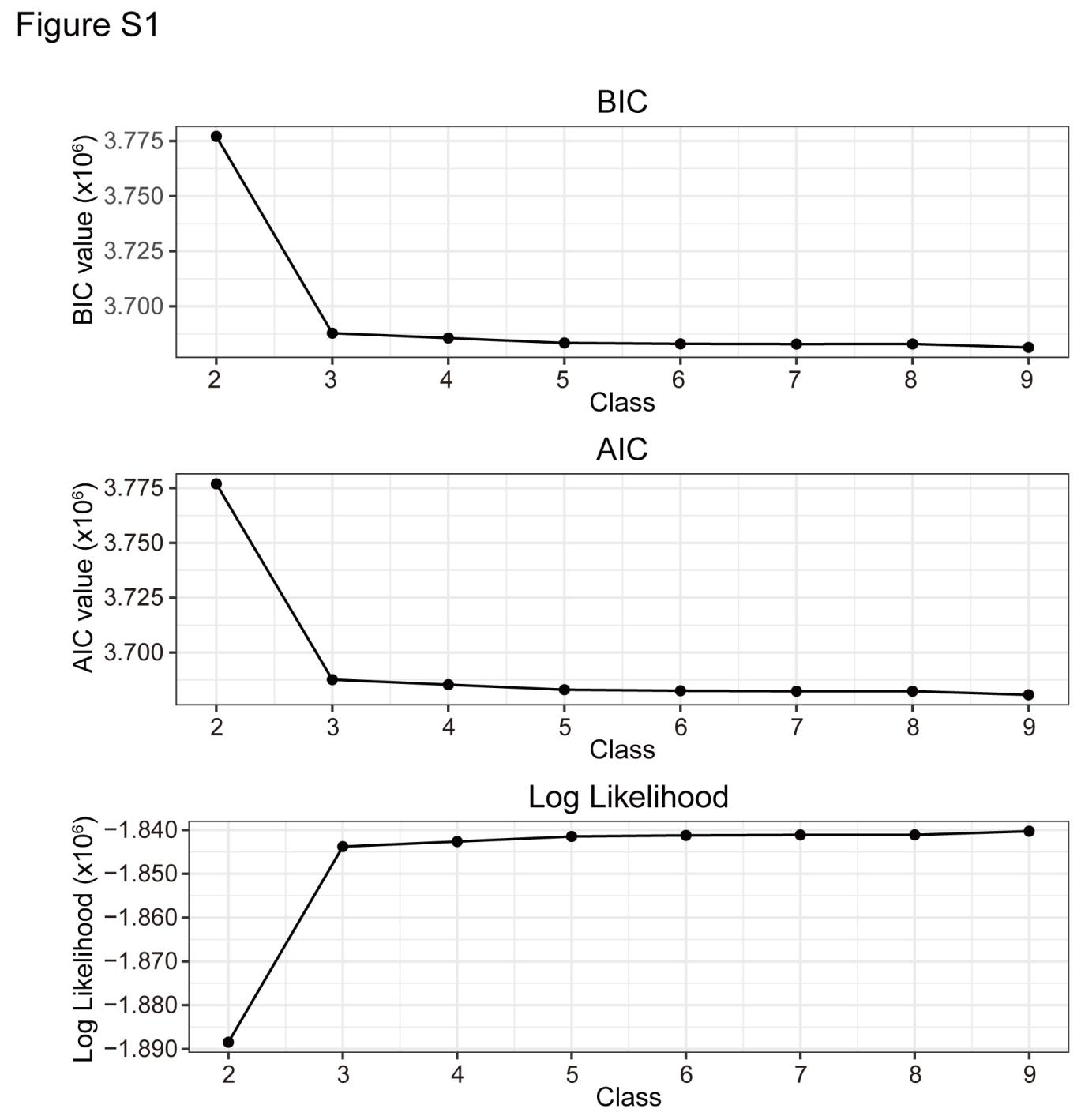


**Figure S1. Scree-like plots of fit statistics (Bayesian Information Criteria (BIC), Akaike Information Criteria (AIC), the log-likelihood (LLIK) ) for latent class models by the number of classes.**
